# Robotic-assisted septal myectomy: Transaortic versus transmitral approaches for hypertrophic obstructive cardiomyopathy

**DOI:** 10.1016/j.xjtc.2026.102289

**Published:** 2026-02-20

**Authors:** Ke Yang, Liu Yu, ChengXin Zhang

**Affiliations:** a Department of Cardiovascular Surgery, First Affiliated Hospital of Anhui Medical University, Hefei, China; b Department of Cardiovascular Surgery, Chizhou First People's Hospital, Chizhou, China

**Figure:**
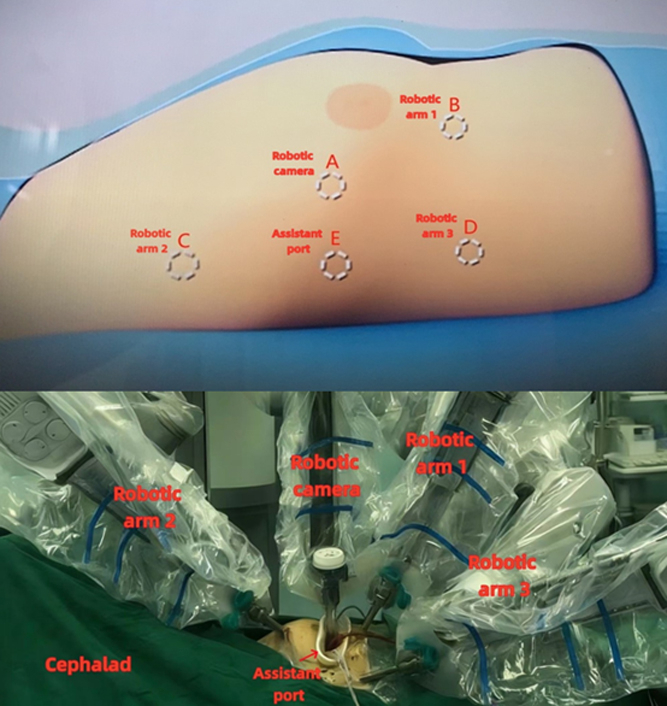
Robotic patient positioning and arm placement for the Morrow procedure.

**Central Message**

Robotic-assisted HOCM myectomy uses transaortic and transmitral approaches. The transaortic approach offers superior efficiency and left ventricular outflow tract reduction for basal hypertrophy. The transmitral approach is for complex mitral regurgitation.

Hypertrophic obstructive cardiomyopathy (HOCM) characterized by left ventricular wall thickening affects approximately 0.6% of adults.^1^ It causes dynamic left ventricular outflow tract obstruction, leading to dyspnea, arrhythmias, heart failure, or sudden cardiac death.^2^ For patients with HOCM with refractory symptoms, septal myectomy (Morrow procedure) is the definitive treatment. This report details 2 distinct robotic-assisted Morrow procedure approaches—transaortic and transmitral—sharing technical experience and highlighting their specific applications in HOCM.

## Material and Methods

### Patient Selection

Patients for robotic-assisted septal myectomy typically have HOCM refractory to medical therapy, a left ventricular outflow tract gradient greater than 50 mm Hg, and clinical symptoms aligning with international guidelines.^3^ The technique choice followed a systematic comparison of approaches (Table 1).

**Table 1:** Robotic septal myectomy: Transaortic versus transmitral approach comparison

Comparison items	Transaortic approach	Transmitral approach
Primary indications	1. Localized or asymmetric hypertrophy of the basal ventricular septum 2. Absence of structural mitral valve pathology requiring intervention (eg, severe degenerative disease, chordal rupture)	1. Septal hypertrophy extending to the mid-ventricle or presenting as diffuse hypertrophy 2. Concomitant mitral valve pathology requiring intervention (eg, SAM-related leaflet elongation, abnormal chordae, hypertrophic or directly fused papillary muscles) 3. Difficult transaortic exposure (eg, small aortic root, severe calcification)
Exposure and resection range	**Advantages:** 1. Direct access to subaortic obstruction 2. Easy localization; avoids manipulation of the mitral valve **Limitations:** 1. Limited exposure of the mid-to-distal septum and apex 2. Difficulty in addressing abnormalities of the subvalvular mitral apparatus	**Advantages:** 1. Excellent exposure of the mid-ventricular to apical septum and the subvalvular mitral apparatus 2. Facilitates concomitant mitral valve procedures (repair/replacement) **Limitations:** 1. Slightly inferior exposure of the basal septum; requires caution to avoid injury to the mitral valve
Impact on mitral valve and SAM	1. Indirectly relieves SAM by alleviating outflow tract obstruction 2. Cannot directly address structural mitral valve pathologies or abnormal chordae/papillary muscles	1. Can directly treat structural mitral abnormalities contributing to SAM (eg, leaflet plication, papillary muscle incision/relocation) 2. Risk of iatrogenic injury to mitral leaflets or chordae during manipulation
Technical challenges	1. Requires precise control of resection depth to prevent septal perforation 2. Must avoid injury to the aortic valve (especially the right coronary cusp) and the conduction system (His bundle located posterior to the membranous septum) 3. Transverse aortotomy needs extension toward the noncoronary sinus for adequate exposure	1. Requires meticulous protection of the anterior mitral leaflet and chordae 2. Resection must avoid injury to the left bundle branch (courses along the left ventricular endocardial surface of the septum)
Perioperative considerations	**Advantages:** 1. No need to open the left atrium, minimizing impact on atrial function 2. Potentially shorter cardiopulmonary bypass time **Risks:** 1. Higher risk of conduction block (especially complete heart block) 2. Risk of aortic valve injury or postoperative regurgitation	**Advantages:** 1. Allows concomitant treatment of mitral valve disease, avoiding reoperation 2. Relatively lower direct risk to the conduction system **Risks:** 1. Risk of mitral repair failure or residual postoperative regurgitation 2. Risks associated with atrial incision (bleeding, thrombosis)
Learning curve and experience requirements	1. Requires familiarity with aortic root anatomy and transaortic instrument angles 2. Recommended after proficiency in robotic aortic valve surgery	1. Requires experience in robotic mitral valve surgery and familiarity with 3D anatomy of the subvalvular apparatus 2. Recommended for centers with extensive experience in open myectomy

*SAM*, Systolic anterior motion; *3D*, 3-dimensional.

### Surgical Technique

**Figure 1:**
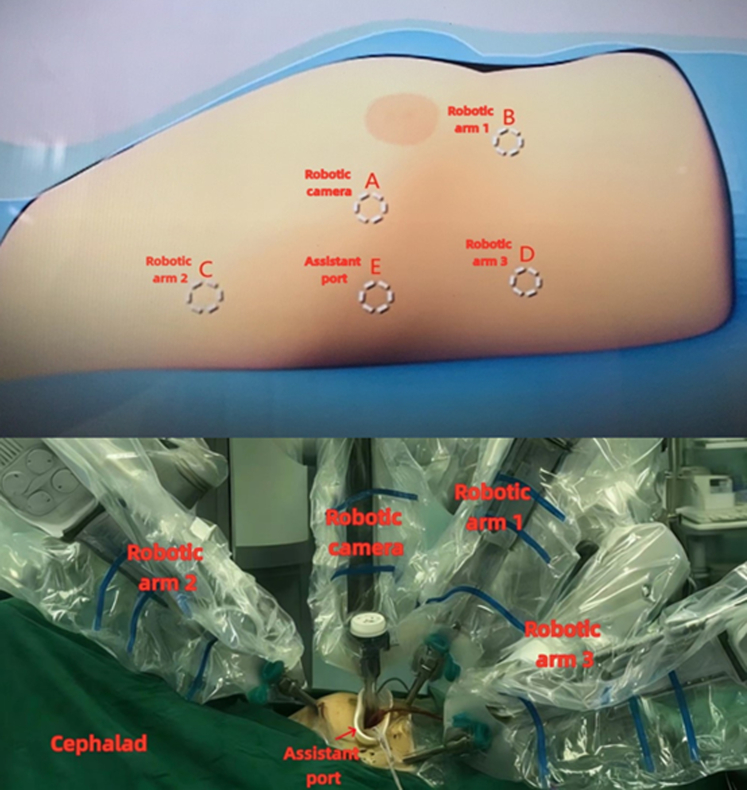
Intraoperative configuration and port placement for robotic-assisted septal myectomy.

Both approaches are performed supine, with right-side elevation and left lung ventilation. The da Vinci camera was positioned at the 4th intercostal space (ICS) (medial to the right anterior axial line). Robotic arms 1, 2, and 3 were placed in the 5th ICS (medial to the right medial collateral ligament), and the 3rd and 6th ICS (medial to the right anterior axial line), respectively (Figure 1).

Resection dimensions were tailored using preoperative transesophageal echocardiography, maintaining a residual septal thickness 10 mm or greater to prevent iatrogenic ventricular septal defect. Resection typically started 5 to 8 mm below the aortic annulus, mainly under the right coronary cusp. A safety margin of 5 mm or more was kept from the membranous septum and the right noncoronary commissure to protect the conduction system.

**Figure 2:**
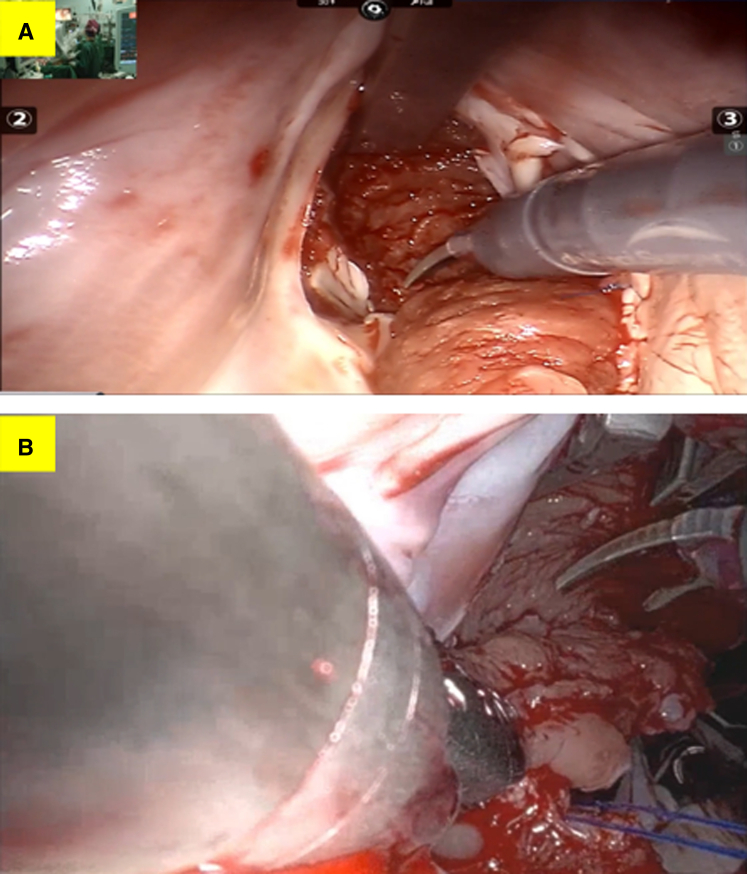
A, Hypertrophied septal myocardium resection via the transmitral approach. B, Hypertrophied septal myocardium resection via the transaortic approach.

Transmitral Approach: Via an interatrial groove incision, the anterior mitral leaflet was incised to enter the left ventricle for myocardial resection (Figure 2, *A*). Anterior leaflet patch reconstruction was routinely performed to prevent the induction or aggravation of mitral regurgitation. Critical steps included precise identification and division of abnormal chordae between the anterior papillary muscle and septum, while meticulously preserving normal chordal structures (Video 1).

Transaortic Approach: A transverse aortotomy was performed and extended 1 to 2 cm toward the noncoronary sinus. This significantly improved exposure of the basal septum and subvalvular apparatus (Video 2). Hypertrophied myocardium beneath the right coronary cusp was then resected under direct vision (Figure 2, *B*).

## Results

Six procedures were successfully completed without thoracotomy conversion or permanent pacemaker implantation (Table 2). The transaortic approach demonstrated superior efficiency, with shorter operative, cardiopulmonary bypass, and crossclamp times. Both reduced left ventricular outflow tract gradients, and the transmitral approach successfully corrected severe mitral regurgitation in all applicable cases.

**Table 2:** Perioperative and postoperative outcomes by surgical approach (mean ± SD)

Parameter	Transaortic (n = 3)	Transmitral (n = 3)
Operative time (min)	280.7 ± 36.0	461.7 ± 46.5
Cardiopulmonary bypass time (min)	152.3 ± 35.4	282.3 ± 31.9
Aortic crossclamp time (min)	93.0 ± 26.0	186.7 ± 12.2
Preoperative IVSD (cm)	**2.10 ± 0.48**	**2.14 ± 0.22**
Preoperative resting LVOT gradient (mm Hg)	**51.3 ± 12.0**	**137.0 ± 12.1**
Postoperative hospital stay (d)	13.3 ± 5.1	28.3 ± 10.4
Postoperative LVEF (%)	65.0 ± 3.6	61.0 ± 2.6
Postoperative IVSD (cm)	1.62 ± 0.58	1.61 ± 0.39
Postoperative LVEDD (mm)	44.9 ± 6.6	48.8 ± 2.6
Postoperative resting LVOT gradient (mm Hg)	16.0 ± 7.8	25.7 ± 10.0
Permanent pacemaker implantation	0	0

Bold values represent newly added data.

*IVSD,* Interventricular septum diastolic; *LVOT,* left ventricular outflow tract; *LVEF,* left ventricular ejection fraction; *LVEDD,* left ventricular end-diastolic diameter.

## Discussion

In the robotic-assisted setting, the approach selection should be based on specific anatomy: The transaortic approach is suitable for isolated basal septal hypertrophy with minimal mitral valve involvement, and the transmitral approach is more appropriate for complex cases involving severe mitral regurgitation, significant systolic aortic motion, subvalvular anomalies, and other similar conditions. Technically, robotic scissors enable precise, multi-angled resection in confined spaces, with 3-dimensional high-definition visualization compensating for the lack of tactile feedback. Continuous irrigation and active suction effectively clear debris.

Drawing from our institutional experience with 37 open myectomies and more than 400 robotic procedures, we believe that although the learning curve for these procedures is steep, it is surmountable for proficient robotic surgery teams. Furthermore, robotic-assisted apical myectomy is a critical alternative for treating mid-ventricular obstruction and apical hypertrophic cardiomyopathy, enabling controlled volumetric reduction to enlarge the left ventricular cavity and enhance stroke volume—the primary goal for improving restrictive apical pathophysiology. Although technically demanding, integrating it into a comprehensive HOCM treatment program can provide a precise, minimally invasive solution for complex obstructions that are difficult to manage with conventional surgery.

### Limitations

Our findings are limited by a small, single-center, retrospective case series (6 patients), precluding statistical significance and generalizability. Potential selection bias and lack of long-term follow-up are also noted.

## Conclusions

Our experience indicates the robotic transaortic approach is highly effective for isolated basal septal hypertrophy, whereas the transmitral approach remains the definitive choice for complex HOCM cases. Both techniques benefit from the precision of robotic instrumentation and should be tailored to the patient's anatomic characteristics.

## Conflict of Interest Statement

The authors reported no conflicts of interest.

The *Journal* policy requires editors and reviewers to disclose conflicts of interest and to decline handling or reviewing manuscripts for which they may have a conflict of interest. The editors and reviewers of this article have no conflicts of interest.

## Supplementary Data

Video 1 Robotic transaortic hypertrophic myectomy. This video demonstrates the surgical details and maneuvers of the hypertrophic myectomy performed via the transaortic approach using the robotic system. Video available at: https://www.jtcvs.org/article/S2666-2507(26)00096-9/fulltext.

Video 2 Robotic transmitral hypertrophic myectomy. This video illustrates the key surgical steps and intraoperative view of the hypertrophic myectomy performed via the transmitral approach with robotic assistance. Video available at: https://www.jtcvs.org/article/S2666-2507(26)00096-9/fulltext.
