# Supplementary figures and images for: Robotic-assisted septal myectomy: Transaortic versus transmitral approaches for hypertrophic obstructive cardiomyopathy
Source: JTCVS Tech. 2026 Feb 20;36:102289. doi: 10.1016/j.xjtc.2026.102289 (PMC13069554; doi:10.1016/j.xjtc.2026.102289)

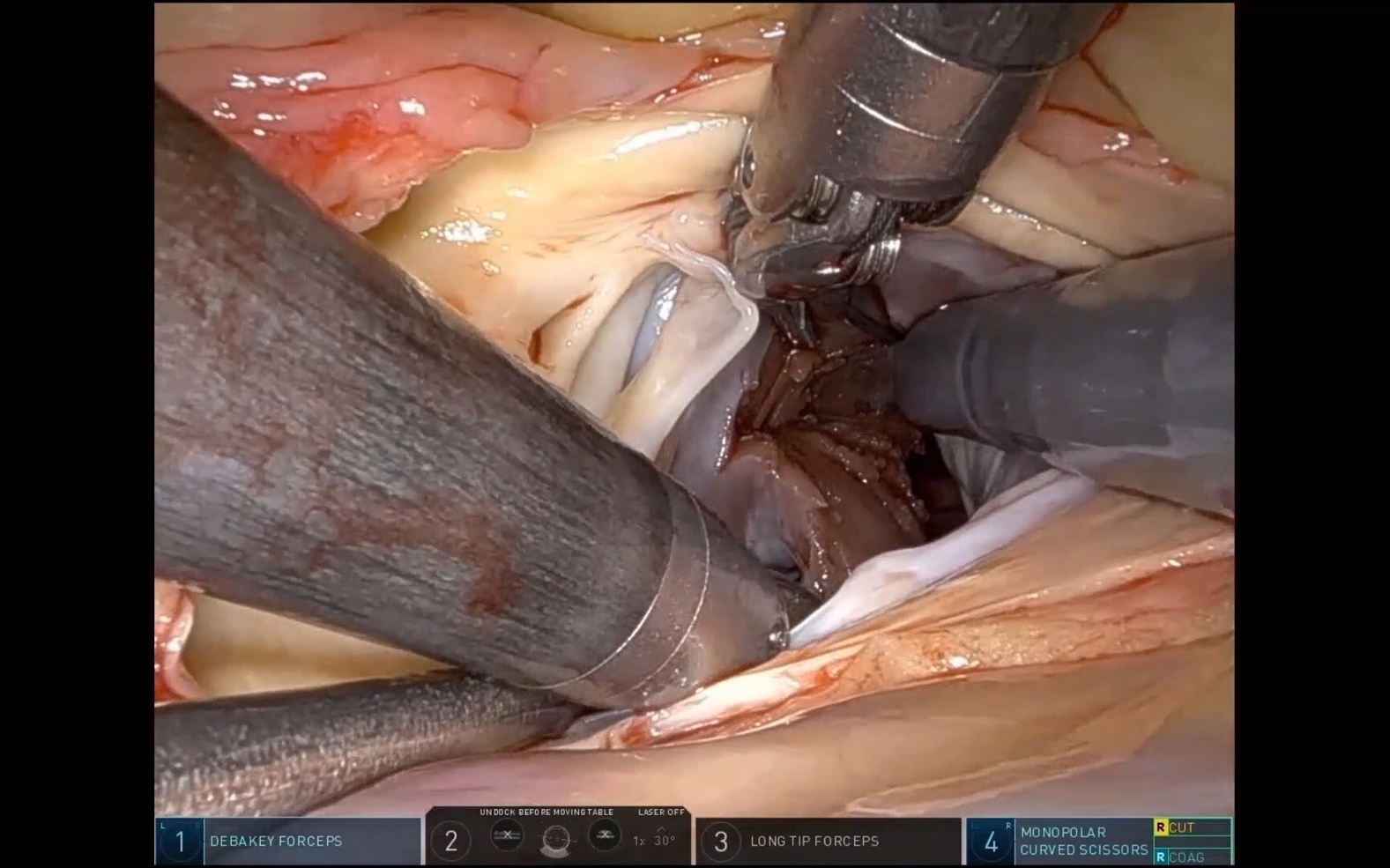

Supplement: Video 1 — Robotic transaortic hypertrophic myectomy. This video demonstrates the surgical details and maneuvers of the hypertrophic myectomy performed via the transaortic approach using the robotic system. Video available at: https://www.jtcvs.org/article/S2666-2507(26)00096-9/fulltext. [file fx2.jpg]

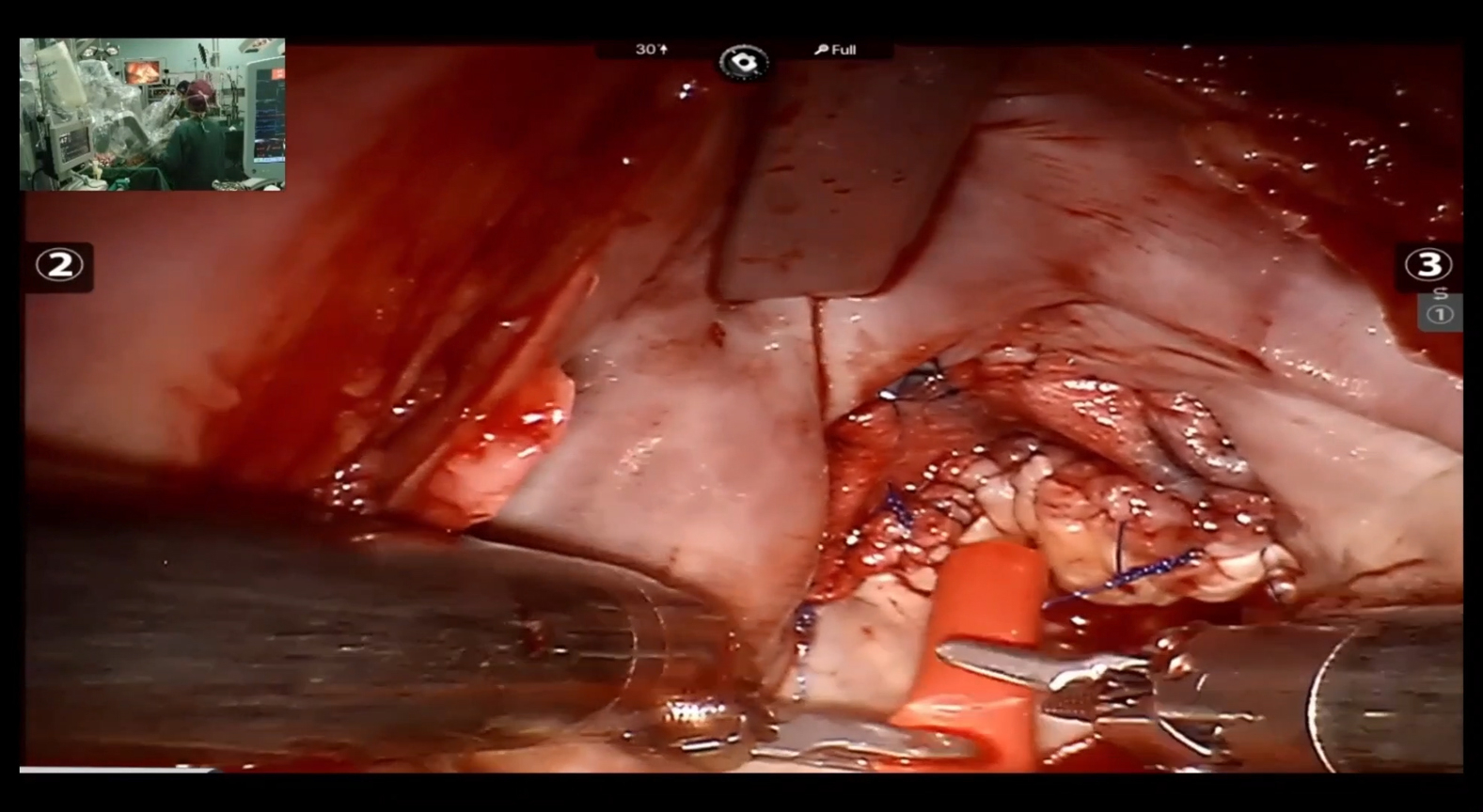

Supplement: Video 2 — Robotic transmitral hypertrophic myectomy. This video illustrates the key surgical steps and intraoperative view of the hypertrophic myectomy performed via the transmitral approach with robotic assistance. Video available at: https://www.jtcvs.org/article/S2666-2507(26)00096-9/fulltext. [file fx3.jpg]
